# Changes in Inflammatory Markers after Administration of Tocilizumab in COVID-19: A Single-Center Retrospective Study

**DOI:** 10.3390/jcm11010107

**Published:** 2021-12-25

**Authors:** Anna Olewicz-Gawlik, Barbara Ginter-Matuszewska, Mikołaj Kamiński, Agnieszka Adamek, Maciej Bura, Iwona Mozer-Lisewska, Arleta Kowala-Piaskowska

**Affiliations:** 1 Department of Infectious Diseases, Hepatology and Acquired Immunodeficiencies, Poznan University of Medical Sciences, ul. Szwajcarska 3, 61-285 Poznan, Poland; bgintermatuszewska@ump.edu.pl (B.G.-M.); mikolaj.w.kaminski@gmail.com (M.K.); agnieszkaadamek@ump.edu.pl (A.A.); mbura@ump.edu.pl (M.B.); iwonalisewska@poczta.onet.pl (I.M.-L.); arletakp1@wp.pl (A.K.-P.); 2 Department of Immunology, Poznan University of Medical Sciences, ul. Rokietnicka 5D, 60-806 Poznan, Poland

**Keywords:** SARS-CoV-2, antiviral, Poland, tocilizumab, COVID-19, interleukin-6

## Abstract

The COVID-19 pandemic requires the development of effective methods for the treatment of severe cases. We aimed to describe clinical outcomes and changes in inflammatory markers in Polish patients treated with tocilizumab. The medical charts of SARS-CoV-2-positive patients treated in the Department of Infectious Diseases between 4 March and 2 September 2020 were retrospectively analyzed. The patients who received tocilizumab according to the Polish Association of Epidemiologists and Infectiologists guidelines were selected for the study. We identified 29 individuals who received tocilizumab, out of whom 11 (37.9%) died. The individuals who died had significantly higher maximal interleukin-6 (IL-6) and lactate dehydrogenase (LDH) serum levels than survivors. After administration of tocilizumab, further increase in LDH and IL-6 was a prognostic factor for unfavorable outcomes. Among inflammatory markers, 7-day mean of IL-6 serum concentration was the best predictor of death (cut-off: ≥417 pg/mL; area under ROC curve = 0.81 [95% Confidence Interval: 0.63–0.98]). The serum concentrations of inflammatory markers before administration of tocilizumab did not predict the outcome, whereas IL-6 and LDH measurements after administration of tocilizumab seemed to be of predictive value.

## 1. Introduction

In December 2019, the first case of severe acute respiratory syndrome coronavirus 2 (SARS-CoV-2) was identified, and the novel coronavirus disease (COVID-19) started to spread rapidly across the world. The outbreak forced physicians to search for effective treatments for patients with a severe course of the disease. In some patients, SARS-CoV-2 infection triggers a cytokine storm and hyperinflammation, leading to multiple organ failure and death [1,2]. Interleukin-6 (IL-6) is one of the key cytokines, playing an important role in the inflammatory reaction, with effects on coagulation cascade, vascular permeability, and myocardial dysfunction leading to tissue hypoxia, hypotension, disseminated intravascular coagulation (DIC), and multiple organ dysfunction [3]. Higher concentrations of IL-6 in serum are associated with higher levels of SARS-CoV-2 viremia [4]. The critical role of IL-6 in the pathophysiology of severe COVID-19 justified its treatment with tocilizumab—a monoclonal antibody against the IL-6 receptor [5]. Tocilizumab was previously used successfully in rheumatoid arthritis, juvenile idiopathic arthritis, giant cell arteritis, and chimeric antigen receptor (CAR)-T-cell-induced cytokine release syndrome [6]. Since the pandemic outbreak, the antibody has been investigated as a potential treatment against severe COVID-19 [7], with contradictory results [8].

In Poland, by the end of 2020, the COVID-19-related overall mortality rate was 2.1% with an 11.5% estimated in-hospital fatality rate for COVID-19 during the first wave of the pandemic [9]. The treatment of COVID-19 with tocilizumab was initiated in April 2020, as the drug was already registered in Poland. Notably, the first experience had been gained before the publication of the first results of RCTs on tocilizumab in COVID-19 [10]. To date, only a few reports have been published on the experience of Polish physicians with this therapy [11,12]. Moreover, evidence on tocilizumab use in COVID-19 patients from randomized trials, nonrandomized trials, and open-label studies are often contradictory [13,14], and there are still limited real-life data about the impact of tocilizumab therapy on the inflammatory markers and survival in COVID-19 patients [15]. Therefore, we aimed to present a single-center experience in COVID-19 treatment using tocilizumab. In this retrospective study, we focused on the clinical outcomes and changes in inflammatory activity in Polish COVID-19 patients treated with tocilizumab.

## 2. Materials and Methods

For this retrospective study, we recruited influenza-negative patients with COVID-19 confirmed by reverse transcription-polymerase chain reaction (RT-PCR), who were hospitalized in the Department of Infectious Diseases, Jozef Strus Multidisciplinary City Hospital, Poznan, Poland (the hospital during the pandemic was transformed into an isolation hospital with a total number of 420 beds, out of which 24 were in the Department of Infectious Diseases), from 4 March 2020 (first COVID-19 case in Poland) to 3 September 2020, and who were treated with tocilizumab, in concordance with the recommendations of the Polish Society of Epidemiology and Infectious Diseases [16,17].

The demographic data and medical history including comorbidities, concomitant therapy, laboratory test results, and clinical outcomes were retrieved from medical records. The Charlson comorbidity index (CCI) was calculated for each patient. Lung involvement was evaluated using chest X-ray and/or computed tomography of the chest (CT). The laboratory tests included white blood cell count (WBC), lactate dehydrogenase (LDH), C-reactive protein (CRP), and IL-6, all of which were assessed at least five times during hospitalization of each patient, using general laboratory equipment and analyzers. Tocilizumab was administered intravenously at 8 mg/kg of body weight (maximally 800 mg) in a single dose, and the second dose was repeated after 8 to 12 h in the absence of improvement, according to the Polish Society of Epidemiology and Infectious Diseases guidelines [16,17], if no contraindication was present. In brief, at the time of the study, tocilizumab treatment was recommended in clinically unstable respiratory failure or acute respiratory distress syndrome patients with COVID-19 with elevated IL-6 concentration, with the repeated dose in the absence of clinical improvement after 8 to 12 h. Contraindications to tocilizumab include known hypersensitivity to tocilizumab or any component of the formulation, and severe, active infection other than COVID-19. Concomitant treatment permitted in the study comprised remdesivir, lopinavir/ritonavir, chloroquine, and glucocorticoids. The exclusion criteria were: active hepatitis B, past hepatitis B virus infection, bacterial sepsis, or tuberculosis suspicion.

IL-6 concentrations were measured using a fully automated Roche Cobas e601 analyzer based on the electro-chemiluminescence immunoassay (ECLIA).

The statistical analysis was performed using the R-programming language 3.6.3 (R Foundation, Vienna, Austria). The results were presented as median (interquartile range) or a number (percentage). The comparisons between the investigated groups were made using the Mann–Whitney *U*-test and chi-squared test, and *p*-value < 0.05 was considered to be significant. Due to the limited number of patients, we did not use the regression analysis. We used the LOESS curve fitting to compare changes in inflammatory markers. The receiver operating characteristic (ROC) curve was analyzed to assess inflammatory marker cut-offs, predicting death after tocilizumab administration. The diagnostic scores were mean concentrations of inflammatory markers (WBC, LDH, CRP, and IL-6) in serum within 7 days before as well as 7 and 14 days after administration of tocilizumab. The classes of observations were: death (coded as 1) and survival (coded as 0). The optimal cut-off point was established using Youden’s index. If the 95% confidence interval of the area under the ROC (AUROC) surpassed 0.50 the association was considered non-significant. The analysis was performed using the ROCit package of R (https://cran.microsoft.com/web/packages/ROCit/ROCit.pdf (accessed on 8 December 2020) [18]).

## 3. Results

Two hundred and seventy-seven patients were hospitalized in the Department of Infectious Diseases in the selected time frame, out of whom 29 patients (20 males and 9 females, median age 58 years) fulfilled the criteria of the Polish Society of Epidemiology and Infectious Diseases for tocilizumab administration (the first infusion of the drug was on 22 April 2020) and were included into the study. Chest X-rays/CT scans revealed features typical of COVID-19 pneumonia, including a ground-glass pattern, in all patients. The general characteristics of the study group are presented in Table 1.

Three out of the twenty-nine (10.3%) patients treated with tocilizumab during hospitalization were also treated with glucocorticoids, lopinavir/ritonavir, and chloroquine, two (6.9%) with glucocorticoids and lopinavir/ritonavir, two (6.9%) with glucocorticoids and chloroquine, two (6.9%) with glucocorticoids, one (3.4%) with glucocorticoids, chloroquine, and remdesivir, and one (3.4%) with lopinavir/ritonavir, chloroquine, and remdesivir. Among patients with arterial hypertension, five (17.2%) received amlodipine, four (13.8%) were treated with sartans, and two (6.9%) with angiotensin-converting enzyme inhibitors; all these patients had favorable outcomes.

Eleven (37.9%) individuals died, and their characteristics are shown in Table 2.

Fourteen patients (48.3%) were admitted to the intensive care unit (ICU), where 12 started tocilizumab treatment, and 10 died. The other members of the investigated group received tocilizumab on the observational ward, with only one fatal outcome. Although the majority of the patients who died had multiple comorbidities and developed multiorgan failure (Table 2), the association of CCI values with clinical outcome did not reach statistical significance (*p* = 0.10). Twenty-three (79.3%) patients received tocilizumab administration twice. A single dose of tocilizumab used in patients ranged from 240 mg to 800 mg. No serious adverse events assigned to the tocilizumab treatment were reported. 

**Figure 1:**
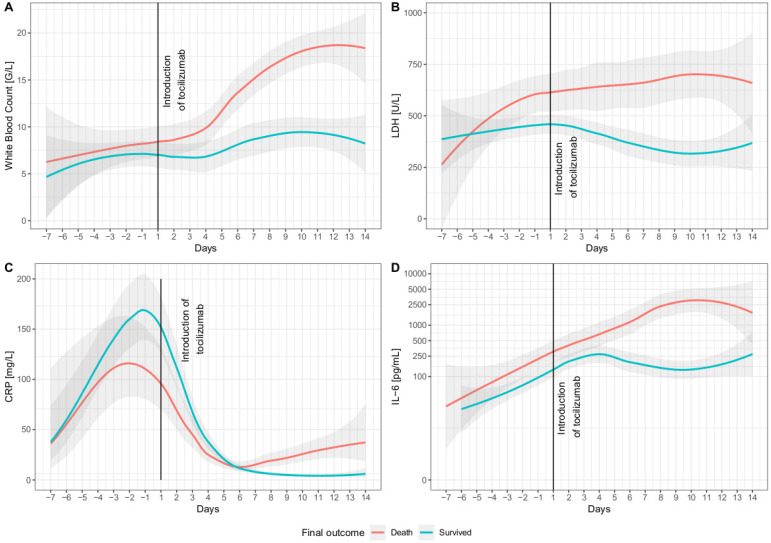
Trends in concentrations of inflammatory markers in serum 7 days before and 14 days after administration of tocilizumab. All charts are presented in linear X-scale, except for chart (**D**), which is presented in log10 scale. (**A**) White blood count; (**B**) lactate dehydrogenase (LDH); (**C**) C-reactive protein (CRP); (**D**) interleukin-6 (IL-6).

The individuals who died commonly reported less cough during admission (Table 1) and had higher maximal IL-6 and LDH serum levels (Figure 1). In contrast, the patients treated with tocilizumab who survived tended to have generally lower levels of WBC, LDH, and IL-6 during the observation period than those who died (Figure 1A,B,D) and were significantly more frequently treated with chloroquine. Interestingly, the CRP serum level (Figure 1C) had higher peaks in survivors than in those with the fatal course of COVID-19. The changes in WBC, LDH, CRP, and IL-6 recorded in the period of 7 days before and 14 days after introducing tocilizumab are shown in Figure 1.

A summary of the ROC analysis is presented in Table 3.

None of the mean serum concentrations of the inflammatory markers measured 7 days before tocilizumab administration proved to be a predictor of the clinical outcome, in contrast to the measurements of WBC, LDH, and IL-6 on days 7 and 14 after the first tocilizumab infusion. The highest predictive value for death or survival was found for IL-6 (7 days after tocilizumab infusion: AUROC = 0.81 (95% CI: 0.63–0.98)) and (14 days after tocilizumab infusion: AUROC = 0.88 (95% CI: 0.74–1.00)), with cut-offs slightly above 400 pg/mL.

## 4. Discussion

In the present study, we investigated the effects of tocilizumab treatment in Polish COVID-19 patients hospitalized in a single center. Tocilizumab emerged as a potential treatment for COVID-19 during the outbreak in Wuhan [15,19]. Results of the first randomized controlled trial, COVACTA, did not show significant improvement in the clinical status of patients treated with tocilizumab [10]. However, the results of two recent meta-analyses showed some evidence that the use of tocilizumab may be associated with a short-term mortality benefit in patients with COVID-19 [13], and with a reduction in the need for mechanical ventilation [13,14]. In our study, the majority of the patients who required tocilizumab had a favorable outcome, and better prognoses were recorded for those who received the drug before admission to the ICU. Gupta et al. also showed that early administration of tocilizumab in the ICU is beneficial in COVID-19 [20]. Similarly, we suspect that a good response was possible thanks to early decisions to administer the drug.

Interestingly, the patients who survived were more likely to receive chloroquine. Hydroxychloroquine during the first wave of COVID-19 was perceived as a potential drug. However, further studies did not confirm its efficacy [21]. Due to the adverse event profile, the antimalarials must be used with caution in patients with heart disease. Therefore, individuals with cardiac comorbidities, who were at higher risk of severe COVID-19, were more likely not to receive chloroquine than the patients without coexisting heart involvement. 

Several risk factors of poor outcome of COVID-19 were identified, such as hypertension, diabetes, obesity, chronic obstructive pulmonary disease, cancer, and cardio- and cerebrovascular disease [22]. Similarly, our results showed that obesity was significantly more frequent in a group of patients who died. However, despite the presence of hypertension in 44.8% of the studied patients, there was no statistical significance between the patients with favorable and fatal outcomes with regard to the high blood pressure, probably due to the small sample size. Interestingly, there were no deaths reported in patients with hypertension who were treated with renin-angiotensin-aldosterone system antagonists or amlodipine. This finding is in agreement with previous observations, which pointed to the beneficial effect of renin–angiotensin–aldosterone system antagonists [23], and amlodipine [24] in improving the clinical outcome of COVID-19 patients. 

The patients who survived vs. those who died did not differ in inflammatory markers serum concentrations on admission. Similarly, mean serum concentrations within 7 days before tocilizumab administration were not associated with the final outcome. This suggests that inflammatory markers before the administration of the drug could not predict the response to tocilizumab. Both trend curves and ROC analysis showed that monitoring IL-6, LDH, and WBC after tocilizumab treatment might help to establish the prognosis. The increase in IL-6 serum concentration after tocilizumab infusion observed in all the studied patients can be explained by diminished receptor-mediated clearance of IL-6 [25]. However, a lack of decrease in IL-6 level 7 days after tocilizumab administration and a further increase in IL-6 and LDH serum concentrations in repeated measurements suggest a lack of response to the treatment and unfavorable outcome. It could also be a signal to change the strategy of treatment. 

The findings of this study must be seen in light of some limitations. Firstly, this is a retrospective analysis of medical charts. Thus, the data collection was not standardized, e.g., the number of laboratory parameter measurements and intervals between them differed among patients. For this reason, we needed to use the LOESS curve fitting to compare changes in inflammatory markers. Secondly, the lack of standardized protocol precludes reliable methodology in reporting adverse events related to tocilizumab. Thirdly, the study population was limited. The hospital had a limited number of analyzed drugs, and tocilizumab was administered only to patients who met the Polish Association of Epidemiologists and Infectiologists criteria [16,17]. For this reason, we could not create a comparable control group. Finally, due to its design, the study does not allow conclusions about the therapy’s efficacy.

To sum up, this is one of few reports on the real-world use of tocilizumab in Poland during the COVID-19 pandemic. We found that monitoring inflammatory markers after but not before administration of tocilizumab may indicate the patients’ further prognosis. Randomized clinical trials on tocilizumab in COVID-19 generated mixed results, and our results suggest that inflammatory marker levels can help to detect individuals who may benefit from tocilizumab therapy.

## 5. Conclusions

More than half of COVID-19 patients who received tocilizumab had a favorable outcome. The measurements of serum levels of IL-6 and LDH after administration of tocilizumab may help to predict the patients’ prognosis.

## Figures and Tables

**Table 1:** General characteristics and comparison of individuals treated with tocilizumab. Data are presented as the median (interquartile range)/*n* (%).

Features	All	Survived	Died	*p*-Value
	*n* = 29 (100.0%)	*n* = 18 (62.1%)	*n* = 11 (37.9%)	
Males (*n*)	20 (69.0%)	13 (72.2%)	7 (63.6%)	0.63
Age (years)	58.0 (53.0–69.0)	55.0 (51.5–62.0)	65.0 (58.0–73.5)	0.13
No comorbidities (*n*)	2 (6.9%)	1 (5.6%)	1 (9.1%)	0.72
Charlson Comorbidity Index (*n*)	2.0 (1.0–4.0)	2.0 (1.0–3.0)	4.0 (2.0–4.0)	0.1
Arterial hypertension (*n*)	14 (44.8%)	10 (55.6%)	4 (36.4%)	0.32
Diabetes (*n*)	7 (24.1%)	3 (16.7%)	4 (36.4%)	0.23
Obesity (*n*)	11 (37.9%)	4 (22.2%)	7 (63.6%)	0.026
Chronic kidney disease (*n*)	4 (13.8%)	2 (11.1%)	2 (18.2%)	0.59
Hypothyroidism (*n*)	2 (6.9%)	2 (11.1%)	0 (0.0%)	0.25
Heart failure (*n*)	1 (3.4%)	0 (0.0%)	1 (9.1%)	0.19
Ischemic heart disease (*n*)	2 (6.9%)	2 (11.1%)	0 (0.0%)	0.25
Atrial fibrillation (*n*)	1 (3.4%)	0 (0.0%)	1 (9.1%)	0.19
Asthma/COPD (*n*)	3 (10.3%)	1 (5.6%)	2 (18.2%)	0.28
Depression (*n*)	1 (3.4%)	0 (0.0%)	1 (9.1%)	0.19
Sclerosis multiplex (*n*)	1 (3.4%)	0 (0.0%)	1 (9.1%)	0.19
Rheumatoid arthritis (*n*)	1 (3.4%)	0 (0.0%)	1 (9.1%)	0.19
Glucocorticoids (*n*)	8 (27.6%)	5 (27.8%)	5 (45.5%)	0.33
Fever (*n*)	25 (86.2%)	17 (94.4%)	8 (72.7%)	0.1
Cough (*n*)	24 (82.8%)	18 (100.0%)	6 (54.5%)	0.02
Dyspnea (*n*)	26 (89.7%)	16 (88.9%)	10 (90.9%)	0.86
Chloroquine (n)	18 (62.1%)	14 (77.8%)	4 (36.4%)	0.026
Lopinavir-ritonavir (*n*)	12 (41.4%)	8 (44.4%)	4 (36.4%)	0.67
Remdesivir (*n*)	2 (6.9%)	1 (5.6%)	1 (9.1%)	0.72
Plasma of convalescents (*n*)	2 (6.9%)	1 (5.6%)	1 (9.1%)	0.72
Stay in ICU (*n*)	14 (48.3%)	3 (16.7%)	11 (100.0%)	<0.001
Intubation (*n*)	12 (41.4%)	1 (5.6%)	11 (100.0%)	<0.001
Length of stay in hospital (*n*)	18.0 (15.0–25.0)	23.0 (18.0–28.2)	15.0 (12.0–17.0)	0.008
Laboratory findings at admission				
WBC (G/L)	6.0 (4.4–8.3)	5.8 (4.1–8.0)	7.3 (4.8–9.6)	0.29
LYMPH (G/L)	0.8 (0.7–1.2)	1.0 (0.7–1.2)	0.8 (0.7–0.8)	0.74
PLT (G/L)	206.0 (172.0–244.0)	204.0 (162.5–234.0)	206.0 (191.0–269.5)	0.44
CRP (mg/L)	104.7 (70.7–182.4)	84.5 (59.5–206.0)	121.4 (87.9–162.9)	0.71
IL-6 (pg/mL)	64.1 (39.8–101.2)	56.9 (40.3–69.7)	82.5 (31.0–322.4)	0.32
Time from admission to tocilizumab administration (days)	4.0 (3.0–7.0)	4.0 (3.2–6.8)	5.0 (2.5–6.0)	0.77
Number of days of treatment by tocilizumab (days)	2.0 (2.0–2.0)	2.0 (2.0–2.0)	2.0 (1.0–2.0)	0.042
Number of tocilizumab treatment (*n*)	2.0 (2.0–2.0)	2.0 (2.0–2.0)	2.0 (1.0–2.0)	0.035

COPD—chronic obstructive pulmonary disease, CRP—C-reactive protein, ICU—intensive care unit, IL-6—Interleukin-6, LYMPH—lymphocytes, PLT—platelets, WBC—white blood cells.

**Table 2:** Characteristics of SARS-CoV-2 infected individuals with the fatal outcome.

No of Patient	Sex	Age (Years)	Comorbidities	Results of the First Blood Culture	Results of the Control Blood Culture	Cause of Death
1.	Female	73	None	*Staphylococcus epidermidis* MSSE	*Acinetobacter baumannii* resistant to carbapenems	Multiorgan failure
2.	Female	83	Depression	*Staphylococcus epidermidis* MSSE	*Acinetobacter baumannii* resistant to carbapenems	Multiorgan failure
3.	Female	88	Atherosclerosis, cerebral aneurysm, osteoporosis, Sclerosis multiplex	Negative	Negative	Respiratory failure
4.	Male	58	Obesity, Chronic Pulmonary Obstructive Disease, Heart Failure	*Staphylococcus epidermidis* MRSE	Negative	Multiorgan failure
5.	Female	70	Asthma, Atrial Fibrillation, Type 2 Diabetes, Rheumatoid Arthritis, Arterial Hypertension, Obesity	Methicillin-resistant coagulase-negative *Staphylococcus* MRCoNS	Not performed	Multiorgan failure
6.	Male	59	Obesity, type 2 diabetes, chronic kidney disease	*Staphylococcus epidermidis* MRSE	Not performed	Multiorgan failure
7.	Male	43	Obesity, arterial hypertension	Negative	Negative	Multiorgan failure
8.	Male	74	Obesity, type 2 diabetes, arterial hypertension	Methicillin-sensitive coagulase-negative *Staphylococcus* MSCoNS	Negative	Multiorgan failure
9.	Male	65	None	Negative	Negative	Respiratory failure
10.	Male	45	Asthma, obesity	Negative	Negative	Sudden cardiac death
11.	Male	58	Type 2 diabetes, Arterial hypertension, hypercholesterolaemia	Methicillin-sensitive coagulase-negative *Staphylococcus* MSCONS	*Acinetobacter baumannii* resistant to carbapenems	Multiorgan failure

MSSE, methicillin-sensitive *Staphylococcus epidermidis*; MRSE, methicillin-resistant *Staphylococcus epidermidis*; MRCoNS, methicillin-resistant coagulase-negative staphylococci, MSCoNS, methicillin-sensitive coagulase-negative staphylococci.

**Table 3:** Results of ROC analysis. The diagnostic scores were mean inflammatory markers serum concentrations, and the classes of observations were: death (coded as 1) and survival (coded as 0).

	Seven Days Before Tocilizumab	Seven Days After Tocilizumab	Fourteen Days After Tocilizumab
White Blood Count (G/L)	Cut-off: ≥9.92 AUROC: 0.55 (95% CI: 0.32–0.79) Sensitivity: 22.2% Specificity: 94.1%	Cut-off: ≥8.40 AUROC: 0.85 (95% CI: 0.70–1.00) Sensitivity: 72.7% Specificity: 88.9%	Cut-off: ≥11.01 AUROC: 0.89 (95% CI: 0.76–1.00) Sensitivity: 90.1% Specificity: 88.9%
Lactate dehydrogenase (U/L)	Cut-off: ≥748 AUROC: 0.63 (95% CI: 0.39–0.87) Sensitivity: 33.3% Specificity: 100.0%	Cut-off: ≥466 AUROC: 0.78 (95% CI: 0.59–0.96) Sensitivity: 90.9% Specificity: 72.2%	Cut-off: ≥493 AUROC: 0.82 (95% CI: 0.65–0.99) Sensitivity: 90.9% Specificity: 83.3%
C-reactive protein (mg/L)	Cut-off: ≥278 AUROC: 0.40 (95% CI: 0.17–0.63) Sensitivity: 11.1% Specificity: 94.1%	Cut-off: ≥167 AUROC: 0.37 (95% CI: 0.17–0.58) Sensitivity: 18.2% Specificity: 94.4%	Cut-off: ≥218 AUROC: 0.44 (95% CI: 0.22–0.66) Sensitivity: 9.1% Specificity: 100.0%
Interleukin-6 (pg/mL)	Cut-off: ≥651 AUROC: 0.59 (95% CI: 0.34–0.83) Sensitivity: 33.3% Specificity: 100.0%	Cut-off: ≥417 AUROC: 0.81 (95% CI: 0.63–0.98) Sensitivity: 81.8% Specificity: 83.3%	Cut-off: ≥425 AUROC: 0.88 (95% CI: 0.74–1.00) Sensitivity: 81.8% Specificity: 83.3%

ROC, receiver operating characteristic; AUROC, area under the receiver operating characteristic.

## Data Availability

The data presented in this study are available in this article.

## References

[1] Huang C., Wang Y., Li X., Ren L., Zhao J., Hu Y., Zhang L., Fan G., Xu J., Gu X. (2020). Clinical features of patients infected with 2019 novel coronavirus in Wuhan, China. Lancet.

[2] Chen N., Zhou M., Dong X., Qu J., Gong F., Han Y., Qiu Y., Wang J., Liu Y., Wei Y. (2020). Epidemiological and clinical characteristics of 99 cases of 2019 novel coronavirus pneumonia in Wuhan, China: A descriptive study. Lancet.

[3] Tanaka T., Narazaki M., Kishimoto T. (2017). Interleukin (IL-6) Immunotherapy. Cold Spring Harb. Perspect. Biol..

[4] Chen X., Zhao B., Qu Y., Chen Y., Xiong J., Feng Y., Men D., Huang Q., Liu Y., Yang B. (2020). Detectable Serum Severe Acute Respiratory Syndrome Coronavirus 2 Viral Load (RNAemia) Is Closely Correlated With Drastically Elevated Interleukin 6 Level in Critically Ill Patients With Coronavirus Disease 2019. Clin. Infect. Dis..

[5] Coomes E.A., Haghbayan H. (2020). Interleukin-6 in COVID-19: A systematic review and meta-analysis. Rev. Med. Virol..

[6] Choy E.H., De Benedetti F., Takeuchi T., Hashizume M., John M.R., Kishimoto T. (2020). Translating IL-6 biology into effective treatments. Nat. Rev. Rheumatol..

[7] Cortegiani A., Ippolito M., Greco M., Granone V., Protti A., Gregoretti C., Giarratano A., Einav S., Cecconi M. (2020). Rationale and evidence on the use of tocilizumab in COVID-19: A systematic review. Pulmonology.

[8] Parr J.B. (2021). Time to Reassess Tocilizumab’s Role in COVID-19 Pneumonia. JAMA Intern. Med..

[9] Kowalska M., Barański K., Brożek G., Kaleta-Pilarska A., Zejda J.E. (2021). COVID-19 related risk of in-hospital death in Silesia, Poland. Pol. Arch. Intern. Med..

[10] Furlow B. (2020). COVACTA trial raises questions about tocilizumab’s benefit in COVID-19. Lancet Rheumatol..

[11] Tomasiewicz K., Piekarska A., Stempkowska-Rejek J., Serafińska S., Gawkowska A., Parczewski M., Niścigorska-Olsen J., Łapiński T.W., Zarębska-Michaluk D., Kowalska J.D. (2020). Tocilizumab for patients with severe COVID-19: A retrospective, multi-center study. Expert Rev. Anti-Infect. Ther..

[12] Podlasin R.B., Kowalska J.D., Pihowicz A., Wojtycha-Kwaśnica B., Thompson M., Dyda T., Czeszko-Paprocka H., Horban A. (2020). How to follow-up a patient who received tocilizumab in severe COVID-19: A case report. Eur. J. Med. Res..

[13] Snow T.A.C., Saleem N., Ambler G., Nastouli E., Singer M., Arulkumaran N. (2021). Tocilizumab in COVID-19: A meta-analysis, trial sequential analysis, and meta-regression of randomized-controlled trials. Intensive Care Med..

[14] Tleyjeh I.M., Kashour Z., Damlaj M., Riaz M., Tlayjeh H., Altannir M., Altannir Y., Al-Tannir M., Tleyjeh R., Hassett L. (2020). Efficacy and safety of tocilizumab in COVID-19 patients: A living systematic review and meta-analysis. Clin. Microbiol. Infect..

[15] Luo P., Liu Y., Qiu L., Liu X., Liu D., Li J. (2020). Tocilizumab treatment in COVID-19: A single center experience. J. Med. Virol..

[16] Flisiak R., Horban A., Jaroszewicz J., Kozielewicz D., Pawłowska M., Parczewski M., Piekarska A., Tomasiewicz K., Zarębska-Michaluk D. (2020). Recommendations of management in SARS-CoV-2 infection of the Polish Association of Epidemiologists and Infectiologists. Pol. Arch. Intern. Med..

[17] Flisiak R., Horban A., Jaroszewicz J., Kozielewicz D., Pawłowska M., Parczewski M., Piekarska A., Simon K., Tomasiewicz K., Zarębska-Michaluk D. (2020). Annex #1 as of 8 June 2020 to: Management of SARS-CoV-2 infection: Recommendations of the Polish Association of Epidemiologists and Infectiologists as of March 31, 2020. Pol. Arch. Intern. Med..

[18] Khan R.A., Brandenburger T. Performance Assessment of Binary Classifier with Visualization. 2019. https://cran.microsoft.com/web/packages/ROCit/ROCit.pdf.

[19] Zhang W., Zhao Y., Zhang F., Wang Q., Li T., Liu Z., Wang J., Qin Y., Zhang X., Yan X. (2020). The use of anti-inflammatory drugs in the treatment of people with severe coronavirus disease 2019 (COVID-19): The Perspectives of clinical immunologists from China. Clin. Immunol..

[20] Gupta S., Wang W., Hayek S.S., Chan L., Mathews K.S., Melamed M.L., Brenner S.K., Leonberg-Yoo A., Schenck E.J., Radbel J. (2021). Association Between Early Treatment with Tocilizumab and Mortality Among Critically Ill Patients With COVID-19. JAMA Intern. Med..

[21] Ghazy R.M., Almaghraby A., Shaaban R., Kamal A., Beshir H., Moursi A., Ramadan A., Taha S.H.N. (2020). Effectiveness and Safety of Chloroquine or Hydroxychloroquine as a mono-therapy or in combination with Azithromycin in the treatment of COVID-19 patients: Systematic Review and Meta-Analysis. medRxiv.

[22] Mason K.E., Maudsley G., McHale P., Pennington A., Day J., Barr B. (2021). Age-Adjusted Associations Between Comorbidity and Outcomes of COVID-19: A Review of the Evidence From the Early Stages of the Pandemic. Front. Public Health.

[23] Chen R., Yang J., Gao X., Ding X., Yang Y., Shen Y., He C., Xiang H., Ke J., Yuan F. (2020). Influence of blood pressure control and application of renin-angiotensin-aldosterone system inhibitors on the outcomes in COVID-19 patients with hypertension. J. Clin. Hypertens..

[24] Zhang L.-K., Sun Y., Zeng H., Wang Q., Jiang X., Shang W.-J., Wu Y., Li S., Zhang Y.-L., Hao Z.-N. (2020). Calcium channel blocker amlodipine besylate therapy is associated with reduced case fatality rate of COVID-19 patients with hypertension. Cell Discov..

[25] Nishimoto N., Terao K., Mima T., Nakahara H., Takagi N., Kakehi T. (2008). Mechanisms and pathologic significances in increase in serum interleukin-6 (IL-6) and soluble IL-6 receptor after administration of an anti–IL-6 receptor antibody, tocilizumab, in patients with rheumatoid arthritis and Castleman disease. Blood.

